# Cerebellar tDCS: How to Do It

**DOI:** 10.1007/s12311-014-0599-7

**Published:** 2014-09-18

**Authors:** Roberta Ferrucci, Francesca Cortese, Alberto Priori

**Affiliations:** 1 Centro Clinico per la Neurostimolazione, le Neurotecnologie e i Disordini del Movimento, Fondazione IRCCS Ca’ Granda Ospedale Maggiore Policlinico, Milan, Italy; 2 Dipartimento di Fisiopatologia Medico Chirurgica e dei Trapianti, Università degli Studi di Milano, Milan, Italy

**Keywords:** Cerebellar tDCS, Electrode position, Polarity, Intensity, Cerebellar stimulation, Ataxia

## Abstract

Cerebellar transcranial direct current stimulation (cerebellar tDCS) is a non-invasive technique for inducing prolonged functional changes in the human cerebellum. Available data show that this simple and safe technique can modulate several motor and non-motor cerebellar functions in healthy humans. Also, preliminary data suggest that cerebellar tDCS is a possible therapeutic option in patients with cerebellar disorders. To provide a reference for those approaching this technique for the first time in healthy humans and patients, we here briefly and practically review the methodology for cerebellar tDCS, discussing electrode types, positions, DC duration and intensity. Recent modelling studies confirm that the electric field generated with the methodology reviewed here reaches the cerebellum at a strength within the range of values for modulating activity in the cerebellar neurons experimentally assessed.

## Introduction

Cerebellar transcranial direct current stimulation (cerebellar tDCS) is increasingly used in neurophysiology laboratories, and its use begins in clinical research [1, 2]. The technique consists in delivering for minutes through a surface scalp electrode a weak (1–2 mA) direct current over the cerebellum. The technique is painless, and stimulation can be delivered during any motor or cognitive activity. Research findings (for a review, see [2]) already provide evidence that cerebellar tDCS can induce neurophysiological changes in the cerebello-brain interaction [3–6] and can influence gait adaptation [7], motor learning [8–12] and cognition [13–18] in healthy humans. Preliminary clinical observations suggest that the changes induced by cerebellar tDCS could be clinically useful in patients with various disorders involving cerebellar dysfunction [19, 20].

Though current evidence leaves open possible (transynaptic or antidromic) changes in other brain or brainstem structures, the physiological effects elicited by cerebellar tDCS arise mainly from functional changes in the cerebellum itself. Cerebellar tDCS could interfere with membrane polarisation in Purkinje cells and in other neurons, fibres (mossy fibres and climbing fibres) and glial cells. DC stimulation applied to the cerebellar cortex in the decerebrated cat influences Purkinje and granular cell activity in a polarity-specific manner; while anodal DC (0.1–1 mA) flowing in the dendrite–axonal direction increases tonic neuronal activity, cathodal DC decreases it [21].

Given the technique’s growing popularity among neuroscientists, for the reader approaching cerebellar tDCS for the first time, we believe it to be useful to describe its methodology. This description has a preliminary limitation; however, insofar, most of the critical methodological variables (for instance, stimulation duration and intensity, number of sessions) have been so far empirically set and no systematic studies have yet assessed how they influence the effects elicited by cerebellar tDCS. Throughout the text, we refer to data available in the literature summarized in Table 1.

**Table 1:**
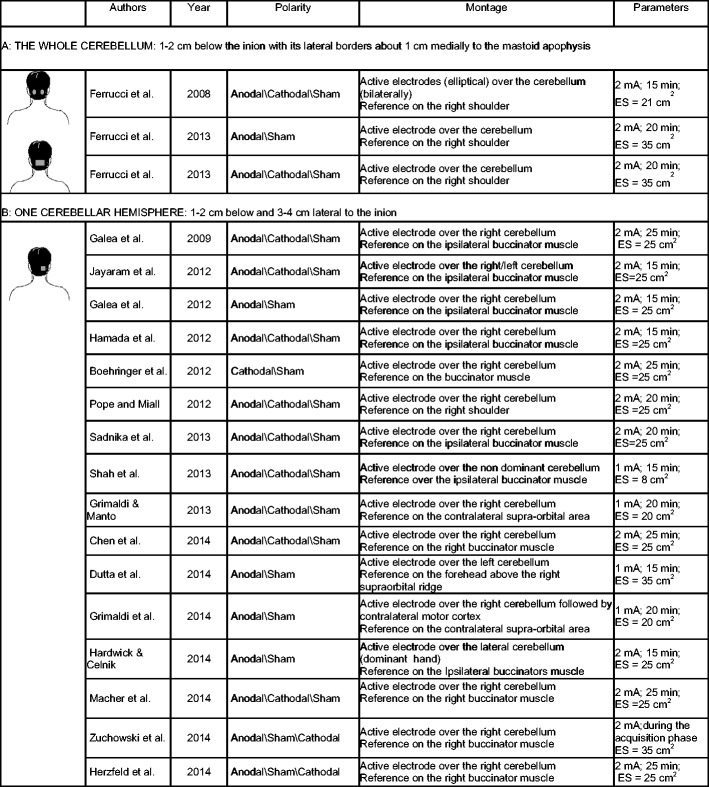
Cerebellar tDCS protocols. Studies with (A) two electrodes over the two cerebellar hemispheres (top) or one electrode over the whole cerebellum (bottom), and (B) electrodes over one cerebellar hemisphere. Note that the position of the reference electrode and stimulation parameters (intensity, duration, electrode size) differed across the various studies

*mA* milliampere, *min* minutes, *ES* electrode size

## Electrodes

Cerebellar tDCS is usually delivered through two rectangular sponge electrodes embedded in a saline-soaked solution (NaCl concentration between 15 to 140 mM) [22]. Electrode size varies; to stimulate half of the cerebellum, most researchers use a single electrode measuring 5 cm × 5 cm (area 25 cm^2^) [3–5, 7, 9–11, 13, 17, 18] while stimulating the whole cerebellum requires a larger electrode measuring about 7 × 5 cm (area 35 cm^2^) [14, 15].

## Electrode Position

The stimulating electrode is placed over the cerebellum and the other (return electrode) over the buccinator muscle [3–13, 18, 23] or the right shoulders [14–17]. The return electrode can also be placed over the scalp [8, 19, 20]. The stimulating electrode can be placed over one or two cerebellar hemisphere (1–2 cm below and 3–4 cm lateral to the inion) [3–13, 16–20, 23] or on the median line over the whole cerebellum (1–2 cm below the inion with its lateral borders about 1 cm medially to the mastoid apophysis) [14, 15] (Table 1).

A key technical point is that because the effects induced by cerebellar tDCS probably depend to a certain extent on the current flow direction and electrical field orientation, the elicited changes depend on the position chosen for the return electrode. For instance, moving the return electrode from the forehead to the ipsilateral cheek over the buccinator muscle changes cerebellar tDCS effects on visuomotor integration [8, 12]. Modelling studies show that with the return electrode over the right shoulder, cerebellar tDCS targets the posterior cerebellum in the adult, with a slight spread to the brainstem in children [24].

After careful skin cleaning, electrodes can be secured in position with an elastic tubular netting or an ergonomic cap. A conductive electrolyte gel can be used between the electrode and the skin (see preceding section). To reduce the risk of burns below the electrodes, electrodes should not be placed over scars, nevi or any other skin abnormalities that could change skin resistance, nor should they be positioned over skull holes or fractures.

## Intensity and Duration

Both electrodes are connected to a standard tDCS stimulator, delivering DC for 15–25 min, at an intensity ranging from 1 to 2 mA. Experimental studies so far reported that the cerebellum has been stimulated with a charge ranging from 0.9 to 2.4 C. This stimulation intensity induces an electric field of the same order of magnitude as that influencing the cerebellar neuron activity in animal experiments [2].

## Polarity of Stimulation

Because cerebellar tDCS has been assessed using different variables (neurophysiological, cognitive, affective, behavioural) with heterogeneous methodologies, interpreting the effects induced by anodal or cathodal stimulation is a far more complex task for cerebellar tDCS than for cerebral tDCS. In essence, when the anodal electrode is placed over the cerebellum, non-motor functions (implicit learning, mismatch negativity) and motor functions in healthy subjects (walking task, visuomotor learning, motor adaptation, eye-blink conditioning, force field learning) and in ataxic patients (tremor and dysmetria) improve. Conversely, when the stimulating electrode is the cathode, memory, split belt walk, paired associative stimulation (PAS), eye-blink conditioning and force field learning worsen in healthy subjects. In some experiments, both polarities induced the same effects. The same polarity with the return electrode placed in a different position could induce different effects [2].

## Adverse Effects

During stimulation in our experience, subjects can perceive a metallic taste and sometimes an itching and tingling sensation below the reference electrode (right shoulders). When cerebellar tDCS ends, subjects often report feeling more motivated and active.

After cerebellar tDCS given within the intensity and duration described here, no subjects have reported adverse effects, nor have patients reported symptoms or signs of cerebellar dysfunction. In most subjects, cerebellar tDCS evokes no sensation probably because cutaneous nerves in the occipital region have a higher threshold than those in the frontal trigeminal dermatomes [25]. If the subject complains of persistent pain or a burning sensation below the tDCS electrodes, the stimulation should be stopped and the skin below the electrodes should be carefully inspected. If there is no redness or lesion, more conductive gel or saline can be added below the electrodes and stimulation can be resumed. If pain or discomfort or both complaints persist, stimulation should be stopped. Because tDCS can spread to the brainstem in children, the technique should be avoided in the paediatric population until systematic and specific safety data are available for children. Other precautions and contraindications are the same as those for cerebral tDCS [26].

## Future Directions

Several methodological variables for cerebellar tDCS remain to be systematically assessed. For example, we need to investigate changes induced by repeated stimulation sessions, compare stimulating electrode montages, examine how body size and age could influence results and study interactions with ongoing drug treatments, the possible effects of random noise or alternating current stimulation and the combined effects of multiple stimulation targets. Multi-target DC stimulation is a fascinating new direction: DC could be used to stimulate the cerebellum, spinal cord and cerebral cortex simultaneously, thus possibly enhancing the induced effects or eliciting still unexplored neuromodulatory responses.
